# The impact of prenatal maternal mental health during the COVID-19 pandemic on birth outcomes: two nested case-control studies within the CONCEPTION cohort

**DOI:** 10.17269/s41997-023-00814-0

**Published:** 2023-09-05

**Authors:** Jessica Gorgui, Vanina Tchuente, Nicolas Pages, Tasnim Fareh, Suzanne King, Guillaume Elgbeili, Anick Bérard, the CONCEPTION Study Group

**Affiliations:** 1 Research Centre, Centre Hospitalier Universitaire Ste-Justine, Montréal, Québec Canada; 2 Faculty of Pharmacy, University of Montreal, Montréal, Québec Canada; 3 Faculty of Medicine, Université Claude Bernard Lyon 1, Lyon, France; 4 Faculty of Medicine, McGill University, Montréal, Québec Canada

**Keywords:** COVID-19 pandemic, Maternal mental health, Pregnancy, Edinburgh Perinatal Depression Scale (EPDS), Generalized anxiety disorders (GAD-7), Stress, Prematurity, Low-birth-weight, Pandémie de la COVID-19, santé mentale maternelle, grossesse, Échelle de dépression postnatale d’Édimbourg (EPDS), Questionnaire d’appréciation des symptômes d’anxiété (GAD-7), stress, prématurité, petit poids à la naissance

## Abstract

**Objective:**

Assess the association between prenatal mental health during the COVID-19 pandemic and preterm birth (PTB; delivery < 37 weeks gestation) and low birth weight (LBW; < 2500 g).

**Methods:**

Pregnant individuals, > 18 years, were recruited in Canada and provided data through a web-based questionnaire. We analyzed data on persons recruited between 06/2020 and 08/2021 who completed questionnaires while pregnant and 2 months post-partum. Data on maternal sociodemographics, comorbidities, medication use, mental health (Edinburgh Postnatal Depression Scale, General Anxiety Disorder-7, stress), pandemic hardship (CONCEPTION—Assessment of Stress from COVID-19), and on gestational age at delivery and birth weight were self-reported. Crude and adjusted odds ratios (aOR) with 95% confidence interval (95%CI) were calculated to quantify the association between PTB/LBW and maternal mental health.

**Results:**

A total of 1265 and 1233 participants were included in the analyses of PTB and LBW, respectively. No associations were observed between PTB and prenatal mental health (depression [aOR 1.01, 95%CI 0.91–1.11], anxiety [aOR 1.04, 95%CI 0.93–1.17], stress [aOR 0.88, 95%CI 0.71–1.10], or hardship [aOR 1.00, 95%CI 0.96–1.04]) after adjusting for potential confounders. The risk of PTB was increased with non-white ethnicity/race (aOR 3.85, 95%CI 1.35–11.00), consistent with the literature. Similar findings were observed for LBW (depression [aOR 1.03, 95%CI 0.96–1.13], anxiety [aOR 1.05, 95%CI 0.95–1.17], COVID stress [aOR 0.92, 95%CI 0.77–1.09], or overall hardship [aOR 0.97, 95%CI 0.94–1.01]).

**Conclusion:**

No association was found between prenatal mental health nor hardship during the COVID-19 pandemic and the risk of PTB or LBW. However, it is imperative to continue the follow-up of mothers and their offspring to detect long-term health problems early.

**Supplementary Information:**

The online version contains supplementary material available at 10.17269/s41997-023-00814-0.

## Résumé

**Objectif:**

Évaluer l’association entre la santé mentale prénatale pendant la pandémie de COVID-19 et les naissances prématurées (accouchement < 37 semaines de gestation) et les faibles poids à la naissance (< 2 500 g).

**Méthodes:**

Des personnes enceintes de plus de 18 ans ont été recrutées au Canada et ont fourni des données prénatales via un questionnaire en ligne. Nous avons analysé les données des personnes recrutées entre 06/2020 et 08/2021, ayant rempli deux questionnaires dont un pendant la grossesse et un 2 mois post-partum. Les données sur les caractéristiques sociodémographiques maternelles, les comorbidités, l’utilisation de médicaments, la santé mentale (Échelle de dépression postnatale d’Édimbourg [EPDS], trouble anxieux généralisé-7 [GAD-7], stress), les difficultés liées à la pandémie (CONCEPTION—Évaluation du stress lié à la COVID-19) ainsi que l’âge gestationnel à l’accouchement et le poids à la naissance ont été auto-déclarées. Les rapports de cotes bruts et ajustés (aRC) avec un intervalle de confiance à 95% (IC 95%) ont été calculés pour quantifier l’association entre la prématurité/petit poids à la naissance et la santé mentale maternelle.

**Résultats:**

Un total de 1 265 et 1 233 participants ont été inclus dans les analyses de NP et de FPN, respectivement. Aucune association n’a été observée entre la prématurité et la santé mentale prénatale (dépression [aRC 1,01, IC 95% 0,91–1,11], anxiété [aOR 1,04, IC 95% 0,93–1,17], stress [aRC 0,88, IC 95% 0,71–1,10], ni difficultés liées à la COVID-19 [aOR 1,00, IC 95% 0,96–1,04]) après ajustement pour les facteurs de confusion potentiels. Le risque de prématurité était plus élevé chez les personnes d’ethnie/race non blanche (aRC 3,85, IC 95% 1,35–11,00), en accord avec la littérature. Des résultats similaires ont été observés pour le faible poids à la naissance (dépression [aRC 1,03, IC 95% 0,96–1,13], anxiété [aRC 1,05, IC 95% 0,95–1,17], stress lié à la COVID [aRC 0,92, IC 95% 0,77–1,09], ou difficultés en lien avec la COVID-19 [aRC 0,97, IC 95% 0,94–1,01]).

**Conclusion:**

Aucune association n’a été trouvée entre la santé mentale prénatale ni les difficultés pendant la pandémie de COVID-19 et le risque de prématurité ou de petit poids à la naissance. Cependant, il est impératif de poursuivre le suivi des mères et de leurs enfants pour détecter précocement d’éventuels problèmes de santé à long terme.

## Introduction

COVID-19 was declared a global pandemic by the World Health Organization on March 11, 2020 (WHO, 2022a), leading to the implementation of several public health measures. In Canada, interventions such as closures of non-essential businesses, social distancing, long-term confinement, and self-isolation were implemented on March 13, 2020 (Canadian Public Health Association, 2021). These interventions impacted pregnant individuals’ health by reducing in-person clinic/hospital visits and access to other health care services and may have intensified stress levels. Many studies, including the CONCEPTION study (Bérard et al., 2022), observed that depression and anxiety scores among pregnant persons during the pandemic were greater compared to pre-pandemic scores (Jones et al., 2022; Wdowiak et al., 2021; Zhao et al., 2022). The CONCEPTION study is a longitudinal cohort that was put in place in June 2020 in response to the pandemic and to the immediate need to better understand the impact of the COVID-19 pandemic on the mental and overall health of pregnant persons as well as its impact on their offspring (Bérard et al., 2022).

External natural events, such as hurricanes, floods, and the COVID-19 pandemic, can contribute to increased populational stress, especially for pregnant persons (Traylor et al., 2020). Exposure to crises can produce short- and long-term health effects on pregnant persons and their offspring (Liu et al., 2016; Marrs et al., 2016). Project Ice Storm, which studied pregnant persons during a crisis leaving a million Quebec/Ontario residents without electricity for up to 45 days, showed that infants’ gestational age was shorter than populational averages, and that other birth outcomes were associated with mothers’ objective levels of hardship from the disaster and their levels of post-traumatic stress (Dancause et al., 2011). Preterm birth (PTB), defined as birth at < 37 weeks of gestation, is the leading cause of neonatal morbidity and mortality (WHO, 2022b). Worldwide, the prevalence of PTB is approximately 11%, ranging from 5% to 18% (WHO, 2018). Reports found no differences in PTB when comparing rates before and during the pandemic (Badran et al., 2021; Jones et al., 2022; Shah et al., 2021; Vaccaro et al., 2021). However, Yang et al. found decreased spontaneous PTB rates (defined as birth at between 22 and 36 weeks of gestation following spontaneous preterm labour or premature rupture of membranes) but not in induced PTB (Yang et al., 2022). In Canada, Shah et al. found no difference in national PTB rates before the pandemic (7.96%) and during the first pandemic year (7.87%, 01/2020–12/2020) (Shah et al., 2021).

Low birth weight (LBW), defined as birth weight < 2500 g, is associated with increased risk of morbidity and mortality (Blencowe et al., 2019). The global LBW prevalence has decreased over time, ranging from 17.5% in 2000 to 14.6% in 2015 (Blencowe et al., 2019). In Canada, LBW rates were 6.3% in 2013 and 6.5% in 2017 (Statistics Canada, 2018). Studies found no differences in LBW rates prior to and during the pandemic (Badran et al., 2021; Jones et al., 2022; Vaccaro et al., 2021). However, rates of extreme LBW decreased significantly (Badran et al., 2021).

There has been much interest in the association between maternal depression, anxiety, and stress during pregnancy and perinatal outcomes such as PTB and LBW, before and since the COVID-19 pandemic; however, results remain controversial. While severe prenatal maternal depression has been associated with an increased risk of PTB (Dowse et al., 2020; Ghimire et al., 2021; Grote et al., 2010; Wdowiak et al., 2021) and LBW (Dowse et al., 2020; Ghimire et al., 2021; Li et al., 2020), other studies did not observe significant associations with PTB nor LBW (Giesbrecht et al., 2022; Jones et al., 2022; Li et al., 2020). As for maternal anxiety, studies did not find a significant association with PTB (Dowse et al., 2020; Giesbrecht et al., 2022; Khoury et al., 2022; Preis et al., 2021), nor with LBW (Dowse et al., 2020; Giesbrecht et al., 2022; Khoury et al., 2022). On the other hand, maternal stress during pregnancy was associated with increased PTB risk (Preis et al., 2021; Zhao et al., 2022). Of note, studies identifying an association between prenatal maternal mental health and adverse perinatal outcomes had large sample sizes or consisted of a meta-analysis grouping a large number of studies, and most used validated tools to measure depression and anxiety.

Given the limited studies and conflicting results on the association between prenatal mental health and perinatal outcomes during the pandemic, we aimed to evaluate PTB and LBW risks associated with prenatal depression, anxiety, stress, and pandemic hardship. The current study is novel due to its objective measures of hardship endured by pregnant persons in the pandemic.

## Methods

### Study design

We conducted two nested case-control studies within the CONCEPTION cohort.

### Setting

The recruitment of pregnant individuals in the CONCEPTION study started on 26/06/2020 and is ongoing. The methodology has been described extensively and published (Bérard et al., 2022; Gorgui et al., 2022; Pagès et al., 2022). In short, Canadian participants were recruited using (i) press releases and media interviews, (ii) social media (e.g. Facebook, Twitter, Instagram, LinkedIn), (iii) in-person in Montreal, and (iv) posters in OB/GYN clinics where links and/or QR codes were posted and directed participants to the survey page. Upon opening the survey page, they first received information on the project, were assessed for eligibility, and then were asked to give consent to participate before moving onto the survey. Questionnaires were made available in English and French; as such, pregnant individuals in Canada who were > 18 years and able to read/understand English or French were eligible.

We obtained individual consent from participants for the baseline and post-partum (2 months) questionnaires. Both questionnaires were completed through SurveyMonkey®. Data were collected online and then downloaded onto a secure server at the *Centre Hospitalier Universitaire Sainte-Justine*, Montréal, Québec. Baseline and 2-months post-partum data were linked by J.G. and analyzed blindly by V.T.

### Participants

We included singleton pregnancies ending with a livebirth for mothers who completed the baseline and the 2-month post-partum questionnaires between 06/2020 and 08/2021 and between 09/2020 and 04/2022, respectively. We excluded multiple pregnancies, as multiplicity is a risk factor for PTB and LBW. In the first nested case-control analysis, we considered PTB to be cases and those with full-term pregnancies to be controls, while in the second we considered LBW to be cases and those born with a weight > 2500 g to be controls. All eligible pregnancies were analyzed.

### Variables

The *baseline questionnaire* collected self-reported data on:

*Maternal characteristics*: (1) sociodemographic characteristics: maternal age at recruitment, years of education, ethnicity/race (Caucasian/white, other), annual household income in Canadian dollars (< C$60,000, C$60,001–$90,000, C$90,001–$120,000, C$120,001–$150,000, and > C$150,000), area of residence (urban, suburban, rural), marital status (living alone (yes/no)), pre-pregnancy height and weight to calculate body mass index (BMI), current number of children (0, 1, ≥ 2), and prenatal care provider (family physician, obstetrician/gynaecologist, midwife, nurse practitioner); (2) maternal lifestyle, including coffee, smoking, alcohol, cannabis, illicit drugs, and multivitamin use (yes/no); (3) maternal history of pregnancy with previous deliveries, abortions, or miscarriage (yes/no); (4) maternal general comorbidities and medication use (including over-the-counter medications).

*Maternal mental health*: (1) Maternal depression was measured using the Edinburgh Postnatal Depression Scale (EPDS) (Shrestha et al., 2016); (2) anxiety was measured using the generalized anxiety disorders scale (GAD-7) (Spitzer et al., 2006); (3) overall maternal stress due to COVID-19 was measured using an item from the Coronavirus Perinatal Experiences Impact Survey (COPE-IS) with a visual analog scale: “What has been your overall level of stress related to COVID-19?”, with response options from 0 (no stress) to 10 (extreme stress) (Lesage et al., 2012; Thomason et al., 2020); and (4) satisfaction with life, measured with a 4-category ordinal scale, with possible responses being “very satisfied”, “satisfied”, “dissatisfied”, and “very unsatisfied” (Statistics Canada, 2023).

We categorized maternal depression and anxiety symptoms scores as follows: no depression < 9, moderate depression 9–12, and severe depression ≥ 13 using the EPDS cut-off (Levis et al., 2020); no anxiety ≤ 9, moderate anxiety 10–15, and severe anxiety > 15 using the GAD-7 cut-off (Spitzer et al., 2006).

*Hardship of pregnant individuals*: The CASC150 (CONCEPTION study Assessment of Stress from COVID-19 – 150 points) is an instrument measuring overall objective hardship experienced throughout the pandemic, and was developed for the CONCEPTION study by S.K. and G.E. It has a maximum score of 150 points and is scored by summing 3 subscales (Threat50, Loss50, Change50), each having a maximal score of 50. Threat50 assesses the level of threat faced due to COVID-19. Loss50 assesses the level of financial loss due to COVID-19. Change50 assesses the amount of change in the daily life and pregnancy plans experienced due to the COVID-19 crisis. Subscale items and measures are further described in Table S1 and Figure S1.

The *2-month post-partum questionnaire* included the self-reported data on:

*Maternal outcomes during pregnancy*: gestational diabetes, gestational hypertension, preeclampsia, bleeding/spotting (yes/no).

*Infant characteristics and perinatal outcomes*: Data included baby’s sex (male/female), gestational age at birth, and birth weight, as well as the following perinatal outcomes (yes/no): COVID-19 diagnosis, small fetal size, neonatal intensive care unit (NICU) or pediatric intensive care unit (PICU) admission, bradycardia, jaundice, extra care required at birth (e.g. resuscitation), and congenital malformation.

### Data analysis

We first compared the means of scores for maternal depression, anxiety, COVID-19 stress, and hardship according to PTB and LBW status. We then calculated the prevalence of the sociodemographic characteristics, maternal outcomes, medical conditions, and medication use during pregnancy according to PTB or LBW status. Finally, we compared infants’ characteristics and perinatal outcomes according to PTB or LBW status. For all descriptive analyses, we calculated standardized mean differences for continuous variables, and chi-square or Fisher’s exact test (when samples in categories were < 5) for categorical variables to conduct the comparisons.

Crude and adjusted odds ratios (OR) with 95% confidence interval (CI) were calculated to determine the risk of PTB and LBW associated with prenatal maternal mental health and hardship during the COVID-19 pandemic, using logistic regression models.

In each crude model, we included maternal mental health (depression, anxiety, overall COVID stress) and maternal hardship. For adjusted models, we added maternal age at recruitment, education, maternal ethnicity, annual household income, area of residence, marital status, pre-pregnancy BMI, care provider (obstetrician/gynaecologist), previous deliveries, maternal lifestyle (coffee intake, smoking, alcohol consumption, cannabis use, multivitamin use), period of recruitment, maternal outcomes during pregnancy (diabetes, hypertension, preeclampsia, bleeding, or spotting), maternal comorbidities (asthma, diabetes, hypertension, nausea, thyroid disease, anemia, chronic migraines, and others), and medication use (including over-the-counter). We have modelized the association between maternal mental health and both outcomes of interest (PTB and LBW) as well as all variables included in adjusted models in Supplemental Fig. 1.

Given the presence of missing data, multiple imputation was performed on the potential covariates listed above based on a linear regression for continuous variables and logistic regression for categorical variables. Statistical analyses were performed using SAS (version 9.4).

### Ethics considerations

The CHU Sainte-Justine’s Research Ethics Committee has approved the study (no. MP-21–2021-2973).

## Results

**Fig. 1:**
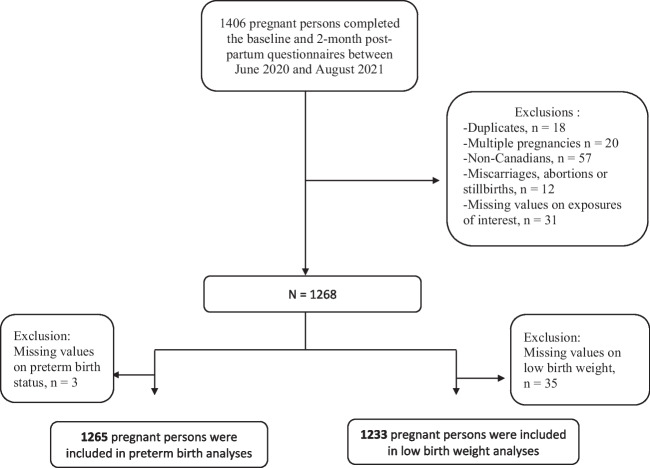
Flow chart of study participants

Overall, 1406 participants answered the baseline and the 2-month post-partum questionnaires. After removing duplicates (*n* = 18); non-Canadian residents (*n* = 57); multiple pregnancies (*n* = 20); miscarriage, stillbirths, or abortions (*n* = 12); and those with missing values on exposures or outcomes of interest (depression, anxiety, COVID-19 stress, hardship, *n* = 31, PTB *n* = 3, LBW *n* = 35), we included 1265 and 1233 participants in the PTB and LBW analyses, respectively (Fig. 1).

Group comparisons by PTB and LBW are presented in Table 1. PTB prevalence was higher among non-Caucasian pregnant persons (6/85 [7.1%] vs 31/1178 [2.6%] *p* = 0.0334) and for pregnant persons followed by an obstetrician/gynaecologist (31/745 [4.2%] vs 7/510 [1.4%] *p* = 0.0046). There were no differences in maternal age at recruitment (*p* = 0.6797), annual household income (*p* = 0.2769), area of residence (*p* = 0.7717), and current number of children (*p* = 0.5013) between PTB and term births. LBW babies were more likely to have an annual household income < C$60,000 (21.8%, LBW vs 9.7%, no LBW) and less likely to have a household income between C$90,001 and $120,000 (9.1%, LBW vs 26.9%, no LBW), and more likely to have a household income between $120,001 and $150,000 (27.3%, LBW vs 19.3%, no LBW) (*p* = 0.0028). There were no differences in maternal age at recruitment (*p* = 0.3596), ethnicity (*p* = 0.0934), area of residence (*p* = 0.3144), marital status/living alone (*p* = 0.6326), and being followed up by an obstetrician/gynaecologist (*p* = 0.1279) between LBW and no LBW (Table 1).

**Table 1:** Sociodemographic characteristics of pregnant women during the COVID-19 pandemic

	Preterm birth status				Low birth weight status			
	Overall *N* = 1265 (%)	Full-term birth *N* = 1227 (97.0%)	Preterm birth^$^ *N* = 38 (3.0%)	*p-value or SMD*	Overall *N* = 1233 (%)	No low birth weight *N* = 1178 (95.5%)	Low birth weight^&^ *N* = 55 (4.5%)	*p-value or SMD*
Maternal age at recruitment, years, mean (SD)	32.6 (4.1)	32.7 (4.1)	32.4 (3.9)	0.01	32.7 (4.0)	32.6 (4.0)	33.2 (4.1)	− 0.12
Education, years, mean (SD)	17.5 (4.3)	17.5 (4.3)	16.5 (3.9)		17.5 (4.3)	17.5 (4.3)	17.8 (3.8)	
Missing	8	8	0	0.23	7	7	0	− 0.08
Ethnicity/race				**0.0334**				0.0934
Caucasian	1178 (93.3)	1147 (93.6)	31 (83.8)		1147 (93.2)	1099 (93.5)	48 (87.3)	
Others	85 (6.7)	79 (6.4)	**6 (16.2)**		84 (6.8)	77 (6.5)	7 (12.7)	
Prefer not to answer	2	1	1		2	2	0	
Annual household income, CAD				0.2769				**0.0028**
< $60,000	124 (10.2)	117 (9.9)	7 (18.4)		122 (10.3)	110 (9.7)	12 (21.8)	
$60,001–$90,000	181 (14.9)	175 (14.9)	6 (15.8)		172 (14.5)	166 (14.7)	**6 (10.9)**	
$90,001–$120,000	312 (25.7)	307 (26.1)	**5 (13.2)**		310 (26.1)	305 (26.9)	**5 (9.1)**	
$120,001–$150,000	238 (19.6)	230 (19.5)	8 (21.0)		233 (19.6)	218 (19.3)	15 (27.3)	
> $150,000	360 (29.6)	348 (29.6)	12 (31.6)		350 (29.5)	333 (29.4)	**17 (30.9)**	
Prefer not to answer	49	49	0		45	45	0	
Missing	1	1	0		1	1		
Area of residence				0.7717				0.3144
Urban	564 (44.8)	549 (45.0)	15 (39.5)		547 (44.6)	517 (44.1)	30 (54.5)	
Suburban	529 (42.0)	512 (41.9)	17 (44.7)		517 (42.1)	498 (42.5)	19 (34.6)	
Rural	166 (13.2)	160 (13.1)	6 (15.8)		163 (13.3)	157 (13.4)	6 (10.9)	
Missing	6	6	0		6	6	0	
Marital status — living alone				0.5786				0.6326
No	1236 (97.8)	1199 (97.8)	37 (97.4)		1204 (97.7)	1149 (97.6)	55 (100.0)	
Yes	28 (2.2)	27 (2.2)	1 (2.6)		28 (2.3)	28 (2.4)	0 (0.0)	
Prefer not to answer	1	1	0		1	1	0	
Pre-pregnancy body mass index, mean (SD)	27.4 (5.7)	27.4 (5.7)	26.4 (4.5)	0.15	27.4 (5.6)	27.4 (5.7)	25.8 (5.0)	0.27
Missing	17	17	0		16	15	1	
Current number of children				0.5013				**0.0228**
0	647 (51.1)	624 (50.8)	23 (60.5)		629 (51.0)	591 (50.2)	38 (69.1)	
1	450 (35.6)	439 (35.8)	11 (29.0)		442 (35.9)	430 (36.5)	**12 (21.8)**	
≥ 2	168 (13.3)	164 (13.4)	4 (10.5)		162 (13.1)	157 (13.3)	5 (9.1)	
Period of recruitment				0.4624				0.2588
June to August 2020	958 (75.8)	927 (75.5)	32 (84.2)		935 (75.8)	898 (76.2)	37 (67.3)	
September 2020 to February 2021	89 (7.0)	87 (7.1)	2 (5.3)		83 (6.7)	79 (6.7)	4 (7.3)	
March to August 2021	217 (17.2)	213 (17.4)	4 (10.5)		215 (17.5)	201 (17.1)	14 (25.4)	
Obstetrician/gynaecologist follow-up				**0.0046**				0.1279
No	510 (40.6)	503 (41.3)	7 (18.4)		499 (40.8)	482 (41.2)	17 (30.9)	
Yes	745 (59.4)	714 (58.7)	**31 (81.6)**		725 (59.2)	687 (58.8)	38 (69.1)	
Missing	10	10	0		9	9	0	

*CAD*, Canadian dollars; *SD*, standard deviation; *SMD*, standardized mean difference

^$^Preterm birth is defined as gestational age at delivery less than 37 weeks

^&^Defined as birth weight at delivery less than 2500 g

Bold numbers indicate significant association (*p* < 0.05)

*p*-values were calculated using chi-square or Fisher test when categories were lower than 5 for categorical variables while standardized mean differences were calculated for continuous variables

Maternal lifestyle during pregnancy, including caffeine, tobacco, alcohol, cannabis, drug, and multivitamin intake, was not different when comparing the term births to PTBs as well as the no LBW compared to LBW (Table 2).

**Table 2:** Maternal history of pregnancy and maternal lifestyle in pregnancy during the COVID-19 pandemic

	Preterm birth status				Low birth weight status			
	Overall *N* = 1265 (%)	Full-term birth *N* = 1227 (97.0%)	Preterm birth^$^ *N* = 38 (3.0%)	*p*	Overall *N* = 1233 (%)	No low birth weight *N* = 1178 (95.5%)	Low birth weight^&^ *N* = 55 (4.5%)	*p*
History of pregnancy								
Previous deliveries				0.1430				**0.0030**
No	651 (51.5)	627 (51.1)	24 (63.2)		633 (51.3)	594 (50.4)	39 (70.9)	
Yes	614 (48.5)	600 (48.9)	14 (36.8)		600 (48.7)	584 (49.6)	**16 (29.1)**	
Previous abortion				0.8025				0.4188
No	1083 (85.6)	1051 (85.7)	32 (84.2)		1055 (85.6)	1010 (85.7)	45 (81.8)	
Yes	182 (14.4)	176 (14.3)	6 (15.8)		178 (14.4)	168 (14.3)	10 (18.2)	
Previous miscarriage				0.4805				0.7582
No	897 (70.9)	872 (71.1)	25 (65.8)		874 (70.9)	834 (70.8)	40 (72.7)	
Yes	368 (29.1)	355 (28.9)	13 (34.2)		359 (29.1)	344 (29.2)	15 (27.3)	
Maternal lifestyle during pregnancy								
Coffee intake				0.1482				0.3000
No	427 (33.8)	410 (33.5)	17 (44.7)		413 (33.6)	391 (33.2)	22 (40.0)	
Yes	836 (66.2)	815 (66.5)	21 (55.3)		818 (66.4)	785 (66.8)	33 (60.0)	
Prefer not to answer	1	1	0		1	1	0	
Missing	1	1	0		1	1	0	
Smoking				0.1193				0.2237
No	1243 (98.4)	1207 (98.5)	36 (94.7)		1211 (98.4)	1158 (98.5)	53 (96.4)	
Yes	20 (1.6)	18 (1.5)	2 (5.3)		2 (1.6)	18 (1.5)	2 (3.6)	
Prefer not to answer	1	1	0		1	1	0	
Missing	1	1	0		1	1	0	
Alcohol consumption				0.3964				0.7190
No	1211 (96.1)	1174 (96.0)	37 (100.0)		1181 (96.2)	1127 (96.1)	54 (98.2)	
Yes	49 (3.9)	49 (4.0)	0 (0.0)		47 (3.8)	46 (3.9)	1 (1.8)	
Prefer not to answer	3	2	1		3	3	0	
Missing	2	2	0		2	2	0	
Use of cannabis products				0.2639				0.0700
No	1254 (99.2)	1217 (99.3)	37 (97.4)		1222 (99.2)	1169 (99.3)	53 (96.4)	
Yes	10 (0.8)	9 (0.7)	1 (2.6)		10 (0.8)	8 (0.7)	2 (3.6)	
Missing	1	1	0		1	1	0	
Use of illicit drugs				**-**				**-**
No	1263 (100.0)	1225 (100.0)	38 (100.0)		1231 (100.0)	1176 (100.0)	55 (100.0)	
Yes	0	0	0		0	0	0	
Missing	2	2	0		2	2	0	
Multivitamin use during pregnancy				0.6125				0.7844
No	152 (12.0)	149 (12.1)	3 (7.9)		149 (12.1)	143 (12.1)	6 (10.9)	
Yes	1113 (88.0)	1078 (87.9)	35 (92.1)		1084 (87.9)	1035 (87.9)	49 (89.1)	

^$^Preterm birth is defined as gestational age at delivery less than 37 weeks

^&^Defined as birth weight at delivery less than 2500 g

Bold numbers indicate significant association (*p* < 0.05)

*p*-values were calculated using chi-square or Fisher test when categories were lower than 5

Table 3 compares term births and PTBs according to maternal mental health and hardship. There were no differences in the prevalence of maternal depression between the term births and PTBs (7.9, SD 5.2 vs 8.2, SD 5.5, respectively; *p* = 0.7560) (Table 3). There were also no differences in mean maternal anxiety between the term births and PTBs (4.2, SD 3.8 vs 4.9, SD 4.4, respectively; *p* = 0.2597); in the mean maternal stress (4.6, SD 2.0 vs 4.4, SD 1.9, respectively; *p* = 0.5426); and in mean hardship (CASC150 – 28.8, SD 8.9 vs 29.5, SD 10.1, respectively; *p* = 0.6513) (Table 3).

**Table 3:** Maternal mental health and resilience during the COVID-19 pandemic

	Preterm birth status				Low birth weight status			
	Overall *N* = 1265 (%)	Full-term birth *N* = 1227 (97.0%)	Preterm birth^$^ *N* = 38 (3.0%)	*p-value or SMD*	Overall *N* = 1233 (%)	No low birth weight *N* = 1178 (95.5%)	Low birth weight^&^ *N* = 55 (4.5%)	*p-value or SMD*
Maternal mental health								
Maternal depression*, mean (SD)	7.9 (5.2)	7.9 (5.2)	8.2 (5.5)	− 0.12	7.9 (5.2)	7.9 (5.2)	8.7 (5.3)	− 0.13
No maternal depression^/^	729 (57.6)	708 (57.7)	21 (55.3)	0.5885	710 (57.6)	683 (58.0)	27 (49.1)	0.2194
Moderate maternal depression	282 (22.3)	275 (22.4)	7 (18.4)		275 (22.3)	263 (22.3)	12 (21.8)	
Severe maternal depression	254 (20.1)	244 (19.9)	10 (26.3)		248 (20.1)	232 (19.7)	16 (29.1)	
Maternal anxiety+, mean (SD)	4.3 (3.8)	4.2 (3.8)	4.9 (4.4)	− 0.18	4.2 (3.9)	4.2 (3.8)	5.0 (4.8)	− 0.21
No maternal anxiety^−^	1157 (91.5)	1122 (91.4)	35 (92.1)	0.8870	1127 (91.4)	1080 (91.7)	47 (85.5)	0.1538
Moderate maternal anxiety	80 (6.3)	78 (6.4)	2 (5.3)		78 (6.3)	72 (6.1)	6 (10.9)	
Severe maternal anxiety	28 (2.2)	27 (2.2)	1 (2.6)		28 (2.3)	26 (2.2)	2 (3.6)	
Maternal stress**, mean (SD)	4.6 (2.0)	4.6 (2.0)	4.4 (1.9)	0.08	4.6 (2.0)	4.6 (2.0)	4.6 (2.1)	0.00
Overall CASC 150^++^, mean (SD)	28.9 (8.9)	28.8 (8.9)	29.5 (10.1)	− 0.07	28.8 (9.0)	28.9 (9.0)	28.2 (8.0)	0.07
Threat50^−−^, mean (SD)	3.7 (4.0)	3.7 (4.0)	4.1 (4.5)	− 0.10	3.7 (4.1)	3.7 (4.1)	3.9 (4.0)	− 0.09
Loss50^%^, mean (SD)	6.6 (5.8)	6.6 (5.7)	7.0 (7.5)	− 0.08	6.6 (5.8)	6.6 (5.9)	6.2 (4.9)	0.06
Change50^//^, mean (SD)	18.6 (4.4)	18.6 (4.4)	18.4 (4.7)	0.04	18.5 (4.4)	18.6 (4.4)	18.1 (4.5)	0.10

*SD*, standard deviation; *SMD*, standardized mean difference

^$^Preterm birth is defined as gestational age at delivery less than 37 weeks

^&^Defined as birth weight at delivery less than 2500 g

^*^Using the Edinburgh postnatal depression scale, continuously

^/^Using the Edinburgh postnatal depression scale cut-off as follows: scale 0 to 8 means no maternal depression, scale 9 to 12 means moderate maternal depression, and scale 13 and more means severe maternal depression

^+^Using the generalized anxiety disorder 7-items scale, continuously

^−^Using the generalized anxiety disorder 7-items scale (GAD-7 cut-off as follows: scale 0 to 9 means no maternal anxiety, scale 10 to 15 means moderate maternal anxiety, and scale 16 and more means severe maternal anxiety)

^**^Using the overall maternal stress related to COVID-19 measured on a scale from 0 (no stress) to 10 (extreme stress)

^++^CASC150 (CONCEPTION study Assessment of Stress from COVID-19 – 150 points) is an instrument measuring the overall objective hardship, or objective stress, experienced by the questionnaire respondent. It has a possible maximum score of 150 points

^−−^Threat50 assesses the level of threat that the respondent faced due to COVID-19. It is measured by items indicating if the health or life of the respondent, or their close ones, was threatened due to COVID-19, e.g. if the respondent or people around them suffered from COVID-19 symptoms and their severity, as well as access to food and medical care

^%^Loss50 assesses the level of financial loss due to COVID-19. It is measured by items indicating if the respondent suffered from a loss of income or savings, job security, insurance, or investments, and how this loss of income affected their life (ability to afford childcare or mortgage)

^//^Change50 assesses the amount of change in the daily life and pregnancy plans that the respondent experienced due to the COVID-19 crisis. It is measured by items indicating changes in daily and work routine, social distancing, pregnancy care support, pregnancy class and care practice, and birth plans, due to COVID-19

Bold numbers indicate significant association (*p* < 0.05)

*p*-values were calculated using chi-square or Fisher test when categories were lower than 5 for categorical variables while standardized mean differences were calculated for continuous variables

There were no differences between no LBW and LBW in maternal depression (7.9, SD 5.2 vs 8.7, SD 5.3, respectively; *p* = 0.2476), maternal anxiety (4.2, SD 3.8 vs 5.0, SD 4.8, respectively; *p* = 0.2286), COVID stress (4.6, SD 2.0 vs 4.6, SD 2.1, respectively; *p* = 0.8898), and COVID-19 hardship (28.9, SD 9.0 vs 28.2, SD 8.0, respectively; *p* = 0.5963) (Table 3).

When considering maternal outcomes during pregnancy, our findings summarized in Table 4 show that there was significantly more preeclampsia among PTBs (10.8%) than among term births (2.6%) (*p* = 0.0194), and more reported bleeding/spotting among PTBs (29.7%) than among term births (13.5%) (*p* = 0.0051). Gestational diabetes was higher among PTBs (22.6%) than among term births (11.1%) (*p* = 0.0106). Medical conditions and medication use were similar between groups except for chronic migraines: they were more prevalent among PTBs (18.4%) than among term births (4.1%) (*p* = 0.0012), as was medication use for chronic migraines (13.5% vs 2.7%, respectively; *p* = 0.0042) (Table 5). No medical conditions or medication use differed significantly between LBW and no LBW (Table 5).

**Table 4:** Maternal outcomes in pregnancy during the COVID-19 pandemic

	Preterm birth status				Low birth weight status			
	Overall *N* = 1265 (%)	Full-term birth *N* = 1227 (97.0%)	Preterm birth^$^ *N* = 38 (3.0%)	*p*	Overall *N* = 1233 (%)	No low birth weight *N* = 1178 (95.5%)	Low birth weight^&^ *N* = 55 (4.5%)	*p*
Gestational diabetes				1.0000				**0.0106**
No	1075 (88.5)	1042 (88.5)	33 (89.2)		1055 (88.4)	1014 (89.9)	42 (77.4)	
Yes	139 (11.5)	135 (11.5)	4 (10.8)		139 (11.6)	127 (11.1)	**12 (22.6)**	
Gestational hypertension				0.7627				0.1883
No	1117 (92.0)	1082 (91.9)	35 (94.6)		1098 (92.0)	1052 (92.2)	46 (86.8)	
Yes	97 (8.0)	95 (8.1)	2 (5.4)		96 (8.0)	89 (7.8)	7 (13.2)	
Preeclampsia				**0.0194**				0.0653
No	1179 (97.1)	1146 (97.4)	33 (89.2)		1159 (97.1)	1110 (97.3)	49 (92.4)	
Yes	35 (2.9)	31 (2.6)	**4 (10.8)**		35 (2.9)	31 (2.7)	4 (7.6)	
Short cervix				1.0000				0.5610
No	1196 (98.5)	1159 (98.5)	37 (100.0)		1176 (98.5)	1124 (98.5)	52 (98.1)	
Yes	18 (1.5)	18 (1.5)	0 (0.0)		18 (1.5)	17 (1.5)	1 (1.9)	
Bleeding or spotting				**0.0051**				0.5459
No	1044 (86.0)	1018 (86.5)	26 (70.3)		1025 (85.8)	981 (86.0)	44 (83.0)	
Yes	170 (14.0)	159 (13.5)	**11 (29.7)**		169 (14.2)	160 (14.0)	9 (17.0)	
Missing	51	50	1		39	37	2	

^$^Preterm birth is defined as gestational age at delivery less than 37 weeks

^&^Defined as birth weight at delivery less than 2500 g

Bold numbers indicate significant association (*p* < 0.05)

*p*-values were calculated using chi-square or Fisher test when categories were lower than 5

**Table 5:** Maternal general comorbidities and medication use during the COVID-19 pandemic

	Preterm birth status				Low birth weight status			
	Overall *N* = 1265 (%)	Full-term birth *N* = 1227 (97.0%)	Preterm birth^$^ *N* = 38 (3.0%)	*p*	Overall *N* = 1233 (%)	No low birth weight *N* = 1178 (95.5%)	Low birth weight^&^ *N* = 55 (4.5%)	*p*
Maternal comorbidities								
Asthma	138 (11.3)	133 (11.2)	5 (13.2)	0.6085	135 (11.3)	125 (11.0)	10 (18.5)	0.0878
Missing	42	42	0		41	40	1	
Asthma medication				0.7523				0.5819
No	1124 (92.7)	1089 (92.8)	35 (92.1)		1095 (92.6)	1048 (92.7)	47 (90.4)	
Yes	88 (7.3)	85 (7.2)	3 (7.9)		87 (7.4)	82 (7.3)	5 (9.6)	
Missing	53	53	0		51	48	3	
Diabetes	57 (4.7)	55 (4.6)	2 (5.3)	0.6960	57 (4.8)	55 (4.8)	2 (3.7)	1.0000
Missing	42	42	0		41	40	1	
Diabetes medication				0.2743				1.0000
No	1188 (97.3)	1152 (97.4)	36 (94.7)		1157 (97.2)	1104 (97.2)	53 (98.2)	
Yes	33 (2.7)	31 (2.6)	2 (5.3)		33 (2.8)	32 (2.8)	1 (1.8)	
Missing	44	44	0		43	42	1	
Hypertension	43 (3.5)	41 (3.5)	2 (5.3)	0.3897	42 (3.5)	40 (3.5)	2 (3.7)	0.7152
Missing	42	42	0		41	40	1	
Hypertension medication				1.0000				1.0000
No	1194 (97.9)	1156 (97.8)	38 (100.0)		1164 (97.8)	1112 (97.8)	52 (98.1)	
Yes	26 (2.1)	26 (2.2)	0 (0.0)		26 (2.2)	25 (2.2)	1 (1.9)	
Missing	45	45	0		43	41	2	
Nausea	261 (21.3)	254 (21.4)	7 (18.4)	0.6554	250 (21.0)	237 (20.8)	13 (24.1)	0.5668
Missing	42	42	0		41	40	1	
Nausea medication				0.8253				0.5831
No	1028 (85.4)	996 (85.5)	32 (84.2)		1005 (85.6)	961 (85.7)	44 (83.0)	
Yes	175 (14.6)	169 (14.5)	6 (15.8)		169 (14.4)	160 (14.3)	9 (17.0)	
Missing	62	62	0		59	57	2	
Thyroid disease	151 (12.4)	147 (12.4)	4 (10.5)	1.0000	148 (12.4)	144 (12.7)	4 (7.4)	0.2533
Missing	42	42	0		41	40	1	
Thyroid medication				1.0000				0.3261
No	1081 (88.5)	1047 (88.4)	34 (89.5)		1053 (88.4)	1003 (88.2)	50 (92.6)	
Yes	141 (11.5)	137 (11.6)	4 (10.5)		138 (11.6)	134 (11.8)	4 (7.4)	
Missing	43	43	0		42	41	1	
Anemia	121 (9.9)	116 (9.8)	5 (13.2)	0.4157	119 (10.0)	112 (9.8)	7 (13.0)	0.4547
Missing	42	42	0		41	40	1	
Anemia medication				0.5290				0.1839
No	1125 (92.3)	1091 (92.4)	34 (89.5)		1096 (92.3)	1049 (92.5)	47 (87.0)	
Yes	94 (7.7)	90 (7.6)	4 (10.5)		92 (7.7)	85 (7.5)	7 (13.0)	
Missing	46	46	0		45	44	1	
Chronic migraines	56 (4.6)	49 (4.1)	**7 (18.4)**	**0.0012**	51 (4.3)	47 (4.1)	4 (7.4)	0.2853
Missing	42	42	0		41	40	1	
Chronic migraines medication				**0.0042**				0.1794
No	1181 (97.0)	1149 (97.3)	32 (86.5)		1154 (97.2)	1104 (97.4)	50 (94.3)	
Yes	37 (3.0)	32 (2.7)	5 (13.5)		33 (2.8)	30 (2.6)	3 (5.7)	
Missing	47	47	0		46	44	2	
Others^/^	325 (26.6)	314 (26.5)	11 (29.0)	0.7365	317 (26.6)	301 (26.5)	16 (29.6)	0.6053
Missing	42	42	0		41	40	1	
Others/^/^ medication				0.7571				0.7581
No	1011 (83.4)	980 (83.5)	31 (81.6)		989 (83.7)	943 (83.6)	46 (85.2)	
Yes	201 (16.6)	194 (16.5)	7 (18.4)		193 (16.3)	185 (16.4)	8 (14.8)	
Missing	53	53	0		51	50	1	
Over-the-counter medication				0.1495				0.5348
No	489 (39.3)	479 (39.7)	10 (27.8)		480 (39.6)	456 (39.4)	24 (43.6)	
Yes	754 (60.7)	728 (60.3)	26 (72.2)		731 (60.4)	700 (60.6)	31 (56.4)	
Missing	22	20	2		22	22	0	

^$^Preterm birth is defined as gestational age at delivery less than 37 weeks

^&^Defined as birth weight at delivery less than 2500 g

^/^Others in high cholesterol, cancer, epilepsy, heart disease, lung disease, ulcer, kidney disease, liver disease, pain, flu, infection, and other diseases

Bold numbers indicate significant association (*p* < 0.05)

*p*-values were calculated using chi-square or Fisher test when categories were lower than 5

Table 6 presents group comparisons according to infant characteristics. There were significantly more males among PTBs (76.3%) than in the term birth group (51.1%) (*p* = 0.0022). Overall, the prevalence of adverse perinatal outcomes (e.g. LBW, small fetal size [*p* = 0.0228], NICU or PICU admission [*p* < 0.0001], jaundice [*p* < 0.0001]) was higher among PTBs compared to term births. Indeed, the prevalence of LBW in the PTB group was 44.7% compared to 3.2% in the term birth, which is expected (*p* < 0.0001). Similarly, the prevalences of all adverse perinatal outcomes were significantly higher among the LBW compared to the no LBW group (Table 6).

**Table 6:** Infants’ characteristics

	Preterm birth status				Low birth weight status			
	Overall *N* = 1265 (%)	Full-term birth *N* = 1227 (97.0%)	Preterm birth^$^ *N* = 38 (3.0%)	*p*	Overall *N* = 1233 (%)	No low birth weight *N* = 1178 (95.5%)	Low birth weight^&^ *N* = 55 (4.5%)	*p*
Infant characteristics								
Baby’s sex				**0.0022**				0.4255
Male	643 (51.9)	614 (51.1)	29 (76.3)		642 (52.1)	611 (51.9)	31 (57.4)	
Female	597 (48.1)	588 (48.9)	**9 (23.7)**		590 (47.9)	567 (48.1)	23 (42.6)	
Missing	25	25	0		1	0	1	
COVID-19 diagnosis				1.0000				0.4180
No	1226 (99.0)	1189 (99.0)	37 (100.0)		1218 (99.0)	1165 (99.1)	53 (98.2)	
Yes	12 (1.0)	12 (1.0)	0 (0.0)		12 (1.0)	11 (0.9)	1 (0.8)	
Missing	27	26	1		3	2	1	
Perinatal outcomes								
Low birth weight^/^	55 (4.5)	38 (3.2)	**17 (44.7)**	** < 0.0001**	**-**	**-**	**-**	**-**
Missing	35	35	0					
Preterm birth^$^	*-*	*-*	*-*	-	38 (3.1)	21 (1.8)	**17 (30.9)**	** < 0.0001**
Missing					1	1	0	
Small fetal size	75 (6.2)	69 (5.9)	**6 (16.2)**	**0.0228**	75 (6.3)	50 (4.4)	**25 (47.2)**	** < 0.0001**
Missing	51	50	1		39	37	2	
NICU or PICU admission	109 (9.3)	87 (7.6)	***22 (59.5)***	** < 0.0001**	108 (9.3)	91 (8.2)	**17 (32.7)**	** < 0.0001**
Missing	87	86	1		68	65	3	
Bradycardia	191 (16.2)	185 (16.2)	6 (16.2)	0.9985	192 (16.5)	179 (16.1)	13 (25.0)	0.0909
Missing	88	87	1		69	66	3	
Jaundice	247 (21.0)	223 (19.6)	**24 (64.9)**	** < 0.0001**	243 (20.9)	223 (20.1)	**20 (38.5)**	**0.0014**
Missing	88	87	1		69	66	3	
Extra care required (e.g. resuscitation)	98 (8.3)	92 (8.1)	6 (16.2)	0.1181	97 (8.3)	88 (7.9)	**9 (17.3)**	**0.0336**
Missing	88	87	1		69	66	3	
Congenital malformation	28 (2.4)	25 (2.2)	3 (8.1)	0.0546	27 (2.3)	23 (2.1)	**4 (7.7)**	**0.0290**
Missing	88	87	1		69	66	3	

*COVID-19*, coronavirus disease 2019; *NICU*, neonatal intensive care unit; *PICU*, pediatric intensive care unit

^$^Preterm birth is defined as gestational age at delivery less than 37 weeks

^/^Defined as birth weight at delivery less than 2500 g

Bold numbers indicate significant association (*p* < 0.05)

*p*-values were calculated using chi-square or Fisher test when categories were lower than 5

As shown in Table 7, after adjusting for all covariates described above, maternal mental health and hardship (namely, depression [aOR 1.01, CI 95% 0.91–1.11], anxiety [aOR 1.04, CI 95% 0.93–1.17], COVID stress [aOR 0.88, CI 95% 0.71–1.10], and overall maternal hardship [aOR 1.00, CI 95% 0.96–1.04]) were not significantly associated with PTB. On the other hand, PTB was significantly associated with preeclampsia (aOR 11.18, CI 95% 2.70–46.38) and gestational bleeding/spotting (aOR 2.98, CI 95% 1.31–6.78) after adjustments. Similarly, PTB was significantly associated with non-white ethnicity (aOR 3.85, CI 95% 1.35–11.00), being followed by an obstetrician/gynaecologist (aOR 2.77, CI 95% 1.12–6.83), the use of any medication (aOR 4.82, CI 95% 1.35–17.24), and presence of chronic migraines (aOR 6.17, CI 95% 1.35–17.24) (Table 7).

**Table 7:** Association between maternal mental health (depression, anxiety, stress, resilience) and the risk of preterm birth

	Full-term birth *N* = 1227 (%)	Preterm birth^$^ *N* = 38 (%)	Unadjusted OR (95%CI)	Adjusted OR^@^ (95%CI)
Maternal depression*, mean (SD)	7.9 (5.2)	8.2 (5.5)	1.01 (0.95; 1.07)	1.01 (0.91; 1.11)
Maternal anxiety^+^, mean (SD)	4.2 (3.8)	4.9 (4.4)	1.04 (0.97; 1.13)	1.04 (0.93; 1.17)
Maternal stress^−^, mean (SD)	4.6 (2.0)	4.4 (1.9)	0.95 (0.81; 1.12)	0.88 (0.71; 1.10)
Maternal CASC 150^++^, mean (SD)	28.8 (8.8)	29.5 (10.1)	1.01 (0.97; 1.04)	1.00 (0.96; 1.04)
Maternal age at recruitment, years, mean (SD)	32.7 (4.1)	32.4 (3.9)	0.98 (0.91; 1.07)	1.00 (0.91; 1.09)
Education, years, mean (SD)	17.5 (4.3)	16.5 (3.9)	0.96 (0.90; 1.02)	0.97 (0.90; 1.05)
Ethnicity/race				
Caucasian/white	1148 (93.6)	32 (84.2)	Reference	Reference
Others^/^	79 (6.4)	6 (15.8)	**2.73 (1.11; 6.71)**	**3.85 (1.35; 11.00)**
Annual household income, CAD				
< $60,000	123 (10.0)	7 (18.4)	Reference	Reference
$60,001–$90,000	181 (14.7)	6 (15.8)	0.58 (0.19; 1.76)	0.49 (0.14; 1.77)
$90,001–$120,000	316 (25.8)	5 (13.2)	**0.28 (0.09; 0.89)**	0.28 (0.08; 1.01)
$120,001–$150,000	237 (19.3)	8 (21.0)	0.59 (0.21; 1.67)	0.65 (0.19; 2.24)
> $150,000	370 (30.2)	12 (31.6)	0.57 (0.22; 1.48)	0.58 (0.18; 1.86)
Area of residence				
Urban	552 (45.0)	15 (39.5)	Reference	Reference
Suburban	515 (42.0)	17 (44.7)	1.22 (0.60; 2.46)	1.48 (0.67; 3.24)
Rural	160 (13.0)	6 (15.8)	1.38 (0.53; 3.62)	1.80 (0.62; 5.26)
Marital status — living alone				
No	1200 (97.8)	37 (97.4)	Reference	Reference
Yes	27 (2.2)	1 (2.6)	1.20 (0.16; 9.08)	1.40 (0.15; 13.22)
Pre-pregnancy body mass index, mean (SD)	27.4 (5.7)	26.4 (4.5)	0.97 (0.91; 1.03)	0.95 (0.89; 1.02)
Obstetrician/gynaecologist care provider				
No	509 (41.5)	7 (18.4)	Reference	Reference
Yes	718 (58.5)	31 (81.6)	**3.14 (1.37; 7.18)**	**2.77 (1.12; 6.83)**
Previous deliveries				
No	627 (51.1)	24 (63.2)	Reference	Reference
Yes	600 (48.9)	14 (36.8)	0.61 (0.31; 1.19)	0.70 (0.32; 1.52)
Coffee intake				
No	411 (33.5)	17 (44.7)	Reference	Reference
Yes	816 (66.5)	21 (55.3)	0.62 (0.33; 1.19)	0.52 (0.25; 1.07)
Smoking				
No	1209 (98.5)	36 (94.7)	Reference	Reference
Yes	18 (1.5)	2 (5.3)	3.73 (0.83; 16.69)	3.81 (0.59; 24.42)
Use of cannabis products				
No	1217 (99.2)	37 (97.4)	Reference	Reference
Yes	10 (0.8)	1 (2.6)	3.29 (0.41; 26.37)	2.34 (0.20; 27.91)
Multivitamin use during pregnancy				
No	149 (12.1)	3 (7.9)	Reference	Reference
Yes	1078 (87.9)	35 (92.1)	1.61 (0.49; 5.31)	1.19 (0.33; 4.32)
Period of recruitment				
June to August 2020	927 (75.5)	32 (84.2)	Reference	Reference
September 2020 to February 2021	87 (7.1)	2 (5.3)	0.67 (0.16; 2.83)	0.40 (0.08; 1.96)
March to August 2021	213 (17.4)	4 (10.5)	0.54 (0.19; 1.56)	0.51 (0.17; 1.58)
Maternal outcomes during pregnancy				
Gestational diabetes	137 (11.2)	4 (10.5)	0.94 (0.33; 2.68)	0.50 (0.11; 2.39)
Gestational hypertension	99 (8.1)	2 (5.3)	0.63 (0.15; 2.67)	0.25 (0.05; 1.39)
Preeclampsia	32 (2.6)	4 (10.5)	**4.39 (1.47; 13.12)**	**11.18 (2.70; 46.38)**
Bleeding or spotting	164 (13.4)	11 (29.0)	**2.64 (1.29; 5.43)**	**2.98 (1.31; 6.78)**
Maternal comorbidities				
Asthma	137 (11.2)	5 (13.2)	1.21 (0.46; 3.14)	1.04 (0.35; 3.09)
Diabetes	56 (4.6)	2 (5.3)	1.16 (0.27; 4.95)	1.25 (0.15; 10.20)
Hypertension	42 (3.4)	2 (5.3)	1.57 (0.37; 6.73)	1.10 (0.19; 6.38)
Nausea	266 (21.7)	7 (18.4)	0.82 (0.36; 1.87)	0.58 (0.23; 1.45)
Thyroid disease	149 (12.1)	4 (10.5)	0.85 (0.30; 2.43)	0.54 (0.17; 1.79)
Anemia	122 (9.9)	5 (13.2)	1.37 (0.53; 3.58)	0.95 (0.33; 2.76)
Chronic migraines	50 (4.1)	7 (18.4)	**5.32 (2.23; 12.66)**	**6.17 (2.20; 17.34)**
Others^&^	328 (26.7)	11 (29.0)	1.12 (0.55; 2.28)	0.84 (0.37; 1.88)
Maternal medication use^**^	938 (76.5)	35 (92.1)	**3.60 (1.10; 11.77)**	**4.82 (1.35; 17.24)**

*OR*, odds ratio; *SD*, standard deviation; *CAD*, Canadian dollars

^$^Preterm birth is defined as gestational age at delivery less than 37 weeks

^@^Adjusted for all the variables in the table

^*^Using the Edinburgh postnatal depression scale (continuous)

+ Using the generalized anxiety disorder 7-items scale (continuous)

–Using the overall maternal stress related to COVID-19 measured on a scale from 0 (no stress) to 10 (extreme stress)

+ + CASC150 (CONCEPTION study Assessment of Stress from COVID-19 – 150 points) is an instrument measuring the overall objective hardship or objective stress experienced by the questionnaire respondent. It has a possible maximum score of 150 points; using 3 subscales: Threat50 assesses the level of threat that the respondent faced due to COVID-19 and is measured by items indicating if the health or life of the respondent/their family was threatened due to COVID-19, e.g. if the respondent or those around them suffered from COVID-19 symptoms and their severity, as well as access to food and medical care. Loss50 assesses the level of financial loss due to COVID-19. It is measured by items indicating if the respondent suffered from a loss of income or savings, job security, insurance, or investments, and how this loss of income affected their life (ability to afford childcare or mortgage). Change50 assesses the amount of change in the daily life and pregnancy plans that the respondent experienced due to the COVID-19 crisis. It is measured by items indicating changes in daily and work routine, social distancing, pregnancy care support, pregnancy class and care practice, and birth plans, due to COVID-19

^/^Other ethnicity in Aboriginal, Asian, Black, Hispanic, other

^&^Others in high cholesterol, cancer, epilepsy, heart disease, lung disease, ulcer, kidney disease, liver disease, pain, flu, infection, and other diseases

^**^Any medication for asthma, diabetes, hypertension, nausea, thyroid disease, anemia, chronic migraines, high cholesterol, cancer, epilepsy, heart disease, lung disease, ulcer, kidney disease, liver disease, pain, flu, infection, other diseases, and over-the-counter medication

Bold numbers indicate significant association (*p* < 0.05)

Table 8 presents the same results for LBW. After adjusting for all the covariates, there were no associations between LBW and maternal mental health (depression [aOR 1.03, CI 95% 0.96–1.13], anxiety [aOR 1.05, CI 95% 0.95–1.17], COVID stress [aOR 0.92, CI 95% 0.77–1.09], or overall hardship [aOR 0.97, CI 95% 0.94–1.01]). Compared to an annual household income < $60,000, there was a significantly lower risk of LBW for women with incomes between $90,001–$120,000 (aOR 0.15, CI 95% 0.05–0.49) and > $150,000 CAD (aOR 0.39, CI 95% 0.16–0.99). In addition, women who had gestational diabetes were significantly more likely to have a baby with LBW (aOR 3.35, CI 95% 1.49–7.55). On the contrary, having already delivered at least one baby was associated with significantly decreased risk of LBW (aOR 0.39, CI 95% 0.20–0.77) (Table 8).

**Table 8:** Association between maternal mental health (depression, anxiety, stress, resilience) and the risk of low birth weight

	No low birth weight *N* = 1178 (%)	Low birth weight^$^ *N* = 55 (%)	Unadjusted OR (95%CI)	Adjusted OR^@^ (95%CI)
Maternal depression*, mean (SD)	7.9 (5.2)	8.7 (5.3)	1.03 (0.98; 1.08)	1.03 (0.96; 1.13)
Maternal anxiety^+^, mean (SD)	4.2 (3.8)	5.0 (4.8)	1.05 (0.99; 1.12)	1.05 (0.95; 1.17)
Maternal stress^−^, mean (SD)	4.6 (2.0)	4.6 (2.1)	1.01 (0.88; 1.15)	0.92 (0.77; 1.09)
Maternal CASC 150^++^, mean (SD)	28.9 (9.0)	28.2 (8.0)	0.99 (0.96; 1.02)	0.97 (0.94; 1.01)
Maternal age at recruitment, years, mean (SD)	32.6 (4.0)	33.2 (4.1)	1.03 (0.97; 1.10)	1.07 (0.99; 1.15)
Education, years, mean (SD)	17.5 (4.3)	17.8 (3.8)	1.02 (0.95; 1.09)	1.02 (0.95; 1.10)
Ethnicity/race				
Caucasian/white	1101 (93.5)	48 (87.3)	Reference	Reference
Others^/^	77 (6.5)	7 (12.7)	2.09 (0.91; 4.76)	2.13 (0.87; 5.26)
Annual household income, CAD				
< $60,000	115 (9.8)	12 (21.8)	Reference	Reference
$60,001–$90,000	172 (14.6)	6 (10.9)	**0.33 (0.12; 0.92)**	0.44 (0.15; 1.31)
$90,001–$120,000	319 (27.1)	5 (9.1)	**0.15 (0.05; 0.44)**	**0.15 (0.05; 0.49)**
$120,001–$150,000	231 (19.6)	15 (27.3)	0.62 (0.28; 1.37)	0.68 (0.28; 1.69)
> $150,000	341 (28.9)	17 (30.9)	0.47 (0.22; 1.00)	**0.39 (0.16; 0.99)**
Area of residence				
Urban	519 (44.1)	30 (54.5)	Reference	Reference
Suburban	501 (42.5)	19 (34.6)	0.66 (0.37; 1.18)	0.76 (0.39; 1.46)
Rural	158 (13.4)	6 (10.9)	0.66 (0.27; 1.61)	0.84 (0.32; 2.20)
Pre-pregnancy body mass index, mean (SD)	27.4 (5.7)	25.9 (5.0)	**0.95 (0.90; 1.00)**	**0.93 (0.88; 0.99)**
Obstetrician/gynaecologist follow-up				
No	485 (41.2)	17 (30.9)	Reference	Reference
Yes	693 (58.8)	38 (69.1)	1.56 (0.87; 2.80)	1.39 (0.73; 2.67)
Previous deliveries				
No	594 (50.4)	39 (70.9)	Reference	Reference
Yes	584 (49.6)	16 (29.1)	**0.42 (0.23; 0.76)**	**0.39 (0.20; 0.77)**
Coffee intake				
No	414 (33.6)	392 (33.3)	Reference	Reference
Yes	819 (66.4)	786 (66.7)	0.75 (0.43; 1.30)	0.73 (0.40; 1.34)
Smoking				
No	1159 (98.4)	53 (96.4)	Reference	Reference
Yes	19 (1.6)	2 (3.6)	2.30 (0.52; 10.14)	3.15 (0.58; 17.19)
Alcohol consumption				
No	1132 (96.1)	54 (98.2)	Reference	Reference
Yes	46 (3.9)	1 (1.8)	0.46 (0.06; 3.37)	0.41 (0.05; 3.48)
Use of cannabis products				
No	1169 (99.2)	53 (96.4)	Reference	Reference
Yes	9 (0.8)	2 (3.6)	**4.90 (1.03; 23.25)**	4.02 (0.59; 27.30)
Multivitamin use during pregnancy				
No	143 (12.1)	6 (10.9)	Reference	Reference
Yes	1035 (87.9)	49 (89.1)	1.13 (0.48; 2.68)	0.98 (0.38; 2.53)
Period of recruitment				
June to August 2020	898 (76.2)	37 (67.3)	Reference	Reference
September 2020 to February 2021	79 (6.7)	4 (7.3)	1.23 (0.43; 3.54)	0.99 (0.32; 3.04)
March to August 2021	201 (17.1)	14 (25.4)	1.69 (0.90; 3.19)	1.68 (0.84; 3.36)
Maternal outcomes during pregnancy				
Gestational diabetes	128 (10.9)	13 (23.6)	**2.54 (1.33; 4.86)**	**3.35 (1.49; 7.55)**
Gestational hypertension	93 (7.9)	7 (12.7)	1.70 (0.75; 3.87)	2.06 (0.72; 5.89)
Preeclampsia	32 (2.7)	4 (7.3)	2.81 (0.96; 8.24)	2.32 (0.64; 8.45)
Bleeding or spotting	164 (13.9)	9 (16.4)	1.21 (0.58; 2.52)	0.99 (0.44; 2.21)
Maternal comorbidities				
Asthma	131 (11.1)	10 (18.2)	1.78 (0.87; 3.61)	1.64 (0.75; 3.60)
Diabetes	56 (4.8)	3 (5.5)	1.16 (0.35; 3.82)	0.38 (0.09; 1.62)
Hypertension	42 (3.6)	3 (5.5)	1.56 (0.47; 5.20)	0.74 (0.17; 3.23)
Nausea	244 (20.7)	13 (23.6)	1.19 (0.63; 2.24)	1.29 (0.63; 2.62)
Thyroid disease	150 (12.7)	4 (7.3)	0.54 (0.19; 1.51)	0.52 (0.17; 1.59)
Anemia	118 (10.0)	7 (12.7)	1.31 (0.58; 2.96)	1.71 (0.71; 4.15)
Chronic migraines	49 (4.2)	5 (9.1)	2.30 (0.88; 6.03)	2.04 (0.67; 6.21)
Others^&^	308 (26.2)	16 (29.1)	1.16 (0.64; 2.10)	0.99 (0.51; 1.94)
Maternal medication use^**^	904 (76.7)	41 (74.6)	0.89 (0.48; 1.65)	0.85 (0.41; 1.76)

*OR*, odds ratio; *SD*, standard deviation; *CAD*, Canadian dollars

^$^Low birth weight (< 2500 g)

^@^Adjusted for all the variables in the table

^*^Using the Edinburgh postnatal depression scale (continuous)

^+^Using the generalized anxiety disorder 7-items scale (continuous)

^−^Using the overall maternal stress related to COVID-19 measured on a scale from 0 (no stress) to 10 (extreme stress)

^++^CASC150 (CONCEPTION study Assessment of Stress from COVID-19 – 150 points) is an instrument measuring the overall objective hardship or objective stress experienced by the questionnaire respondent. It has a possible maximum score of 150 points; using 3 subscales: Threat50 assesses the level of threat that the respondent faced due to COVID-19 and is measured by items indicating if the health or life of the respondent/their family was threatened due to COVID-19, e.g. if the respondent or those around them suffered from COVID-19 symptoms and their severity, as well as access to food and medical care. Loss50 assesses the level of financial loss due to COVID-19. It is measured by items indicating if the respondent suffered from a loss of income or savings, job security, insurance, or investments, and how this loss of income affected their life (ability to afford childcare or mortgage). Change50 assesses the amount of change in the daily life and pregnancy plans that the respondent experienced due to the COVID-19 crisis. It is measured by items indicating changes in daily and work routine, social distancing, pregnancy care support, pregnancy class and care practice, and birth plans, due to COVID-19

^/^Other ethnicity in Aboriginal, Asian, Black, Hispanic, other

^&^Others in high cholesterol, cancer, epilepsy, heart disease, lung disease, ulcer, kidney disease, liver disease, pain, flu, infection, and other diseases

^**^Any medication for asthma, diabetes, hypertension, nausea, thyroid disease, anemia, chronic migraines, high cholesterol, cancer, epilepsy, heart disease, lung disease, ulcer, kidney disease, liver disease, pain, flu, infection, other diseases, and over-the-counter medication

Bold numbers indicate significant associations (*p* < 0.05)

## Discussion

In this study, we assessed the associations between the risk of preterm birth and low birth weight and prenatal mental health (e.g. depression, anxiety, and overall pandemic-related stress) and hardship during the COVID-19 pandemic. The prevalences of PTB and LBW among our cohort were 3.0% (*n* = 38) and 4.5% (*n* = 55), respectively. After adjustments, we observed no significant associations between prenatal mental health and the risk of PTB and LBW. PTB was significantly associated with known predictors such as non-white ethnicity, having an obstetrician/gynaecologist care provider, preeclampsia, gestational bleeding/spotting, chronic migraines, or any medication use during pregnancy, which is reassuring. Gestational diabetes was associated with increased LBW risk. After adjustments by potential confounders, decreased LBW risk was significantly associated with an annual household income between C$90,001 and $120,000 or > C$150,000 (vs < $60,000), higher pre-pregnancy BMI, and previous live births.

Although information on the impact of prenatal maternal mental health such as depression, anxiety and stress, as well as hardship during the COVID-19 pandemic remains limited, our results are consistent with a number of published studies. In fact, Li et al. (2020) showed no significant association between depression and PTB. No associations between maternal mental health and PTB/LBW were observed in Wales (United Kingdom), despite a similarly high impact of the pandemic on mental health (Jones et al., 2022). As for anxiety during pregnancy, no significant association with PTB was found (Preis et al., 2021). A number of studies failed to find an association between anxiety and PTB or LBW (Dowse et al., 2020; Giesbrecht et al., 2022; Khoury et al., 2022). Overall, we observed that results are considerably heterogeneous as two studies report significantly increased risk of PTB and LBW associated with maternal stress (Preis et al., 2021; Zhao et al., 2022) and maternal depression (Grote et al., 2010; Li et al., 2020) while others have shown that greater maternal depression is associated with PTB and LBW (Dowse et al., 2020; Wdowiak et al., 2021). These differences in results compared to ours could be explained by the fact that we used different questionnaires to assess maternal mental health. In fact, two studies also using the EPDS, a validated tool to measure depression, did not identify any associations between prenatal maternal depression and the risk of PTB (Li et al., 2020). However, Dowse et al. (2020) found lower gestational age at birth associated with prenatal depression using the EPDS, which could be attributed to their large population compared to ours (*n* = 53,646). As for maternal anxiety, one study assessing anxiety using GAD-7 did not find a significant association between prenatal anxiety and PTB (Preis et al., 2021).

Our data included a new measure of hardship from the COVID-19 pandemic based on relatively objective self-reported items: the CASC150. The total score, and its three subscales (Threat50, Loss50, and Change50), had small-to-moderate associations with greater maternal depression and anxiety, with reductions in life satisfaction since the onset of the pandemic, and with greater perceived stress due to the pandemic. However, neither the total score nor any of the subscales were significant predictors of preterm birth or low birth weight. It is also important to note that the CASC150 has not yet been validated. The failure to find an association between the severity of exposure to the pandemic and birth outcomes is consistent with a recent study that used administrative population data on 147,349 births: birth outcomes (birth weight, LBW, gestational age at birth and PTB) did not differ for pregnancies that were exposed to the 1998 Quebec ice storm compared to pregnancies in the 3 years before and the 3 years after the crisis (Project Ice Storm) (Ahmed et al., 2022). As well, birth outcomes were also unrelated to the estimated duration of power outages for each pregnancy that had been exposed to the storm. If exposure to this severe stressor and the severity of that exposure were not associated with birth outcomes in a large population study, then it is not surprising that no associations were found in the current cohort. However, it is important to note that despite not finding an increased risk of adverse perinatal outcomes in association with maternal mental health, later Project Ice Storm results suggest that child development was impacted by maternal mental health in the long term (Dancause et al., 2011).

### Strengths and limitations

Our study is one of the first studies with a longitudinal follow-up to assess maternal and perinatal outcomes during the COVID-19 pandemic. Moreover, our study used a multi-methods recruitment strategy which allowed us to recruit effectively across Canada. Our data were collected online, thus accelerating the speed at which the study was performed and allowing us to access real-time data and results, which is essential in the context of an ever-evolving pandemic. In addition, our study is the first to use a fairly objective measure, the CASC150, to assess different aspects of maternal prenatal hardship during the COVID-19 pandemic and the risk of perinatal outcomes. Although the collected data was self-reported, we used validated tools to measure depression (EPDS), anxiety (GAD-7), and stress using a visual analog 10-point scale commonly used in the clinical setting (Lesage et al., 2012) as well as the quality of life indicator used by Statistics Canada (Statistics Canada, 2023).

In our study, we had a lower rate of PTB (3%) compared to the rate in Canada (7.8%), giving us relatively low numbers of PTB (*n* = 38) and LBW (*n* = 55), and rendering our study underpowered (4.8% and 25.8%, respectively — post hoc power calculation). Despite a lengthy questionnaire (≈20 min for the baseline and ≈15 min for the 2-month post-partum), we collected many variables of interest with a high completion rate (85% for both the baseline and the 2-month post-partum). This allowed us to adjust for a number of confounding variables when quantifying the risk of PTB/LBW in association with maternal mental health. This said, we cannot rule out unmeasured confounding, namely history of PTB/LBW which would further bias our results towards the null. Furthermore, our population has a higher annual income than the general Canadian population. In fact, the median household income in 2020 was $104,350 CAD whereas the median salary in our study was between $120,000 and $150,000 (Statistics Canada, 2022). However, access to the study to pregnant individuals was provided across social media groups as well as community clinics, which provide access to individuals with lower socioeconomic status. Given the online recruitment strategy, we cannot rule out the introduction of a selection bias where individuals more concerned with the COVID-19 pandemic would have been included preferentially in the study. However, as we do not have the denominator nor any information on non-participants, this is difficult to assess. This said, it is possible that given their higher socioeconomic status, higher level of education, and low exposure to substance use, our participants are at a lower risk of PTB/LBW, which would affect the generalizability of our findings to a similar population.

## Conclusion

We did not find associations between prenatal depression, anxiety, COVID stress, and pandemic hardship during the COVID-19 pandemic and the risk of PTB and LBW. However, given that the COVID-19 pandemic has had an important effect on increased scores of maternal mental health and the importance of the burden of maternal depression/anxiety/stress, and given the findings from the Project Ice Storm, it is imperative to continue the follow-up of mothers and their offspring to detect any long-term health problems at an early stage prior to school entry.

## Contributions to knowledge

What does this study add to existing knowledge?

- Our study is among the first with a longitudinal follow-up to assess maternal and perinatal outcomes during the COVID-19 pandemic.
- Our study uses a multi-method recruitment strategy with data collected online, thus accelerating the speed at which the study was performed and allowing us to access real-time data, which is essential in the context of an ever-evolving pandemic.
- Our study introduces an in-depth assessment of the hardship from the COVID-19 pandemic that uses relatively objective items assessing levels of threat, loss, and change.
- Though our findings did not demonstrate a statistically significant association, our study gives an opportunity to continue the follow-up of mothers and their offspring in order to detect early any long-term health problems.

What are the key implications for public health interventions, practice, or policy?

- Our study suggests that public health interventions, practice, and policy need to provide adequate prenatal mental health care to mitigate the impact on maternal and perinatal outcomes.
- Medical practice and public health interventions should be aware of higher rates of mental health problems among pregnant persons during the COVID-19 pandemic (Bérard et al., 2022) in order to maximize resources in mental health care and thus provide multiple strategies to reduce the burden on mental health during pregnancy (e.g. medications, physical activity, social support).

## Supplementary Information

Below is the link to the electronic supplementary material.

Supplementary file1 (DOCX 71 KB)

Supplementary file2 (DOCX 35 KB)

## Data Availability

Anonymized individual-level data from the study including data dictionaries, data collection tools, and codes will be made available upon request. Requests for access will be reviewed by a data access committee.
