# Supplementary material for: The impact of prenatal maternal mental health during the COVID-19 pandemic on birth outcomes: two nested case-control studies within the CONCEPTION cohort
Source: Can J Public Health. 2023 Sep 5;114(5):755–73. doi: 10.17269/s41997-023-00814-0 (PMC10485209; doi:10.17269/s41997-023-00814-0)
Supplement: Supplementary file 1 — Supplementary file1 (DOCX 71 KB) [file 41997_2023_814_MOESM1_ESM.docx]

**Supplemental figure 1.** Directed acyclic graph modelling the association between maternal mental health and both outcomes of interest, preterm birth (PTB) and low birth weight (LBW).

Legend: BMI – body mass index, LBW – low birth weight, PTB – preterm birth.
